# Abdominal wall endometriosis: a case report

**DOI:** 10.1093/jscr/rjab055

**Published:** 2021-04-06

**Authors:** Stefanos K Stefanou, Kostas Tepelenis, Christos K Stefanou, George Gogos-Pappas, Christos Tsalikidis, Konstantinos Vlachos

**Affiliations:** Department of Surgery, General Hospital of Ioannina, G. Chatzikosta, Ioannina, Greece; Department of Surgery, University Hospital of Ioannina, Ioannina, Greece; Department of Surgery, General Hospital of Ioannina, G. Chatzikosta, Ioannina, Greece; Department of Surgery, University Hospital of Ioannina, Ioannina, Greece; Second Department of Surgery, University General Hospital of Alexandroupoli, Alexandroupoli, Greece; Department of Surgery, University Hospital of Ioannina, Ioannina, Greece

**Keywords:** abdominal wall, endometriosis, endometrioma, ultrasound, fine-needle biopsy

## Abstract

Abdominal wall endometriosis has an incidence of 0.3–1% of extrapelvic disease. Α 48-year-old female appeared in the emergency department with cellulitis in a lower midline incision. She had an endometrioma of the anterior abdominal wall removed 2 years ago. After 5 months, she underwent an open repair of an incisional hernia with a propylene mesh, which was unfortunately infected and removed 1 month later. Finally, in July 2019, she had her incisional hernia repaired with a biological mesh. Imaging modalities revealed a large mass below the umbilicus. Mass was punctured under ultrasound guidance. Cytology reported the recurrence of endometriosis. Pain and abdominal mass associating with menses were the two most typical symptoms. Wide local excision of the mass with at least 1 cm negative margins is the preferred treatment. Surgeons should maintain a high suspicion of the disease in reproductive women with circular pain, palpable abdominal mass and history of uterine-relating surgery.

## INTRODUCTION

Endometriosis is a common condition where endometrial cells, both glands and stroma, are found outside the womb [1]. Most often, endometriosis is located on the ovaries, fallopian tubes and tissue around the uterus and ovaries, whereas the extrapelvic disease is rare [2]. Abdominal wall endometriosis (AWE), the commonest site of extrapelvic disease, has an incidence of 0.03–1% [3]. Cause is not entirely clear, and several theories have been proposed about its pathogenesis [2, 4]. The main symptom is a recurrent cyclic pain associated with menstruation [5]. Differential diagnosis encompasses hernias, abscesses, lipomas, desmoids tumors and malignancies [6].

## CASE REPORT

A 48-year-old female patient visited the emergency department due to cellulitis in a lower midline incision. She had a tumor of the anterior abdominal wall removed at 2017, which turned out to be an endometrioma in the histological report. Six months later, she underwent an open repair of an incisional hernia with a polypropylene mesh. The mesh was placed on lay. One month after the surgery, the mesh was removed owing to infection. On July 2019, her incisional hernia was repaired with a biological mesh. From obstetric history, she had a caesarian delivery 18 years ago.

Abdominal examination disclosed a palpable hard mass below the umbilicus. The patient was admitted to the surgical department for observation, and antibiotics were commenced, particularly vancomycin. A computed tomography (CT) of the abdomen was obtained, which demonstrated a large oval mass 10.7 × 5.7 × 7.8 cm with rim enhancement and dense content, which was located below the umbilicus.

On the same day, percutaneous drainage of the mass was carried out. Macroscopically, the fluid seemed to be blood. Then, a small drainage catheter was left in place to drain the hematoma.

Inflammation of the anterior abdominal wall was subsided on Day 2, while the percutaneous drainage catheter was removed on Day 3. The culture was sterile, and the patient was discharged on Day 8 without complications. Cytology report evinced the diagnosis of AWE: the presence of endometrial glands and stroma.

## DISCUSSION

Endometriosis is the presence of endometrial mucosa, both glands and stroma, outside the uterus [1]. Its incidence in the general population is 6–10% and it usually affects reproductive women. However, the exact incidence of endometriosis is difficult to capture as the diagnosis requires biopsy or visual identification of the endometrium during laparoscopy or laparotomy [7].

AWE was first reported by Meyer in 1903 [8]. It is the most common site of extrapelvic disease with an incidence of 0.03–1% [3, 8]. Although the exact pathogenesis remains unclear, several theories have been proposed. Direct transplantation theory postulates that endometrial cells can be transported to the abdominal wall during surgery involving the uterine cavity, such as hysterectomy or cesarean delivery. Coelomic metaplasia theory suggests that cells in the mesothelial lining of the abdominal peritoneum can differentiate themselves into endometrial cells. It seems that hormonal and immunological factors stimulate this procedure. Finally, lymphatic and vascular metastasis theory proposes that endometrial cells enter circulation and are deposited at the abdominal wall [2, 4].

The most frequent appearance is a cyclic pain associated with menstruation. Other symptoms include skin discoloration, bleeding, dysmenorrheal, dyspareunia and bowel or bladder symptoms, whereas some patients have no symptoms. Clinical examination reveals a palpable abdominal mass, which is usually immobile and painful. Even though pain, skin discoloration, bleeding and an increase in the size of the mass are linked with menses, only 57% of the patients exhibit cyclic symptoms [5].

Abdominal ultrasound is regarded as the first-line imaging modality for masses and mass-like lesions in the abdominal wall. AWE is depicted as a heterogeneous hypoechoic mass with irregular shape and indistinct margins. It is usually solid, though sometimes has a cystic appearance. Occasionally, vascularity can be seen on color Doppler imaging [6, 9].

CT and magnetic resonance imaging (MRI) of the abdomen provide useful information for choosing the best method for closing the fascia defect during operation, as they reveal the extent of the disease and the involvement of the fascia of the rectus muscle. The imaging findings on CT encompass a solid soft-tissue heterogeneous mass with mild-to-moderate enhancement after the administration of intravenous contrast material. On MRI, it is portrayed as a hyperintense or isointense heterogeneous lesion on both T1- and T2-weighted images. Feeding vessels can be observed on occasion on both imaging modalities [6, 9].

Fine needle aspiration under ultrasound guidance is an easy, inexpensive and accurate procedure to confirm the diagnosis of AWE. Caution should be taken to avoid the introduction of new implants at the puncture sites. In case of a limited amount of sample material, an additional biopsy may be necessary [6, 8].

AWE needs to be distinguished preoperatively from hernias, abscesses, lipomas, desmoids tumors and malignancies. It is worth noting that the malignant transformation of endometriosis is rare, with an incidence of 1% of cases [9].

The treatment of choice for AWE is wide local excision of the mass with at least 1 cm negative margins [7, 10]. Mesh reconstruction should be taken into account for patients with an abdominal mass ≥5 cm on ultrasound or with the involvement of the abdominal wall fascia and muscle [5, 8]. The reported recurrence rate is approximately 5% [8, 10]. Higher recurrence rates are associated with large lesions and involvement of the rectus muscle [10]. Medical treatment is ineffective in treating AWE. The recurrence rate is high, especially after the cessation of the drug [7, 8].

## CONCLUSION

Pain and abdominal mass associating with menses are the two most typical symptoms of AWE. Abdominal ultrasound is the first-line modality for the diagnosis of AWE, while CT and MRI disclose the extent of the disease and the involvement of rectus muscle. Wide local excision with negative margins of 1 cm at least is the preferred treatment. Surgeons should maintain a high suspicion of the disease in reproductive women with circular pain, palpable abdominal mass and history of uterine-relating surgery.

## CONFLICT OF INTEREST STATEMENT

None declared.

## FUNDING

None.

## CONSENT FOR PUBLICATION

Written informed consent was obtained from the patient prior to publication.
